# The need for measurable targets in national action plans on antimicrobial resistance: opportunities for action

**DOI:** 10.3389/fpubh.2026.1875275

**Published:** 2026-09-03

**Authors:** Uswa H. Shafaque, Arne Ruckert, Samuel Orubu, Kayla Strong, Suzanne Garkay Naro, Susan Rogers Van Katwyk, Mathieu J. P. Poirier

**Affiliations:** 1 AMR Policy Accelerator, Global Strategy Lab, Toronto, ON, Canada; 2 School of Global Health, Faculty of Health, York University, North York, ON, Canada

**Keywords:** antimicrobial resistance, health policy, national action plans, surveillance, target setting

## Abstract

Antimicrobial resistance (AMR) represents a significant global health challenge that defies simple solutions and requires multisectoral coordination and interdisciplinary collaboration. National action plans (NAPs), which are built on the template of the World Health Organization’s Global Action Plan on AMR, offer a critical framework for addressing AMR. However, the lack of measurable targets within many NAPs hinders progress in mitigating AMR and setting up accountability and monitoring frameworks for tracking implementation progress. In this perspective article, we argue that establishing national targets within NAPs will be a critical step towards the effective mitigation of AMR. We selectively review the current landscape of targets in existing NAPs, reflect on the importance of establishing context-specific AMR targets and identify early successes in AMR target development. Finally, we present recommendations for developing clear, measurable, and achievable targets in NAPs to improve AMR outcomes. We finally suggest that success requires not only strong political will but also sustained investments in integrated surveillance infrastructure to facilitate multi-sectoral policy coordination and collaboration in target setting processes.

## Background

Antimicrobial resistance (AMR) poses a significant threat to global health, with low-income countries (LICs) disproportionately affected by its various impacts. Recent modelling estimates suggest that close to 5 million deaths annually are associated with AMR, as well as over 1 million deaths directly attributable to bacterial AMR (1). In the Global Action Plan (GAP) on AMR, the World Health Organization (WHO) called upon member states to develop and implement national action plans (NAPs) on AMR, using coordinated actions across relevant One Health sectors. In 2024, the international community also committed to a set of targets and actions to address AMR, including reducing the global death burden associated with AMR annually by 10% by 2030. However, while some countries have successfully implemented national AMR targets, most of the existing NAPs lack concrete deliverables, clear timelines, and measurable outcomes, indicating that more global guidance is needed to integrate targets into NAPs. Even though broad goals are important in setting the strategic policy direction, policy interventions can be difficult to assess without measurable indicators and clear targets to assess a country’s progress in AMR mitigation. This article reviews some of the existing efforts to integrate targets into NAPs and argues that integrating clear targets will improve global AMR mitigation efforts by strengthening accountability and facilitating better monitoring and evaluation through cross-sectoral coordination.

## Measurable targets for AMR mitigation

Targets are a central aspect of effective AMR policy implementation as they facilitate the operationalization of specific, time-bound, and measurable objectives set within a policy or strategy. Developing time-bound targets allows broad policy ambitions to be turned into practical actions. Thus, numerical and time-bound targets can support achieving broader goals (i.e., qualitative aims that describe an intended outcome without specific metrics), and targets allow monitoring and evaluation of progress and effectiveness of specific AMR interventions. Targets also establish a framework for accountability by clearly defining success and enabling stakeholders to track commitments, identify gaps, and take corrective action when goals are not met. To follow best practices, targets should also be time-bound and aligned with overarching policy goals, such as reducing inappropriate antimicrobial use (AMU), decreasing AMR prevalence, or improving surveillance systems. In their absence, NAPs risk becoming aspirational documents rather than actionable strategies (2).

In the African region, targets in NAPs, when present, are mostly disaggregated – associated either with NAP objectives or discreet activities, and listed in operational plans, monitoring and evaluation (M&E) frameworks, or process indicator matrices. Malawi summarizes key AMR targets for the five NAP objectives, and lists quantitative annual output targets, or indicators, for specific activities described under these strategic objectives (3). Ghana’s NAP targets are mostly both qualitative and activity-specific. Liberia and Namibia’s NAPs contain numeric targets for some activities under M&E as target, or impact, respectively. These examples illustrate, at best, a hierarchy of target definitions (4–6). Exceptions include Ethiopia with a target 10% reduction in resistant blood-stream infections by 2025, in its 3rd NAP on AMR.

In the Asian region, some countries have implemented measurable targets related to AMU. For example, Japan’s NAP on AMR was developed in 2016 and introduced specific targets for AMU reduction by 2020 relative to 2013, including a 30% reduction in total AMU, and a 50% reduction for broad-spectrum antibiotics (7). This has allowed Japan to experience significant reductions in AMU as it was able to focus its resources on strategic actions to achieve key milestones. Analysis of annual AMU trends in Japan from 2016 to 2020 shows a general decline in the use of oral antimicrobials, especially those classified in the Watch category (9.69 DID in 2019 to 7.22 DID in 2020) (7). In Thailand, a 20% reduction in antimicrobial drug consumption in humans and a 30% reduction in antimicrobial drug consumption in animals between 2017 and 2022 have been established as national targets, with the animal health target having been achieved by 2021 (8).

Similarly, in the European region, the Danish government introduced the yellow card scheme in 2010 for the livestock sector, spelling out specific AMU reduction targets and use thresholds for punishment in case of non-compliance (9). This resulted in a significant and immediate reduction in just 1 year among high-consumption weaner pig herds, peaking at an 82% reduction in January 2011, with further declines observed among sows and finishers after legislative changes in 2014. This suggests that specific targets linked to the yellow card scheme in Denmark drove a sustained reduction in AMU in animal production from 2010 to 2020 (9). These examples demonstrate that having specific, quantifiable targets can drive meaningful improvements in reducing AMU, which in turn can help to mitigate AMR.

However, it is important to recognize that many of the first-generation NAPs were adapted from the WHO GAP on AMR, which itself did not recommend inclusion of concrete target. This suggests that the limited presence of numerical benchmarks also reflects historical gaps in global guidance rather than local lack of technical capacity or political will. This is why targets should become a focus in the updated (2026) Global Action Plan on AMR as more global guidance is needed on target development. In the next section, we provide some recommendations that could inform global reflections on appropriate national level AMR targets.

## Recommendations for target setting

To support effective AMR policymaking, countries must prioritize developing context-specific strategies for target setting, addressing five key challenges:

### Develop context-specific collaborative strategies for target setting

Target development should follow a collaborative process, and apply a multi-sectoral approach, as can be seen in our discussion of how Denmark developed targets in this article. This process may involve policymakers strengthening existing multisectoral collaboration mechanisms, building on existing national AMR steering committees, intersectoral committees, or task forces with clearly defined roles, responsibilities, as well as integrating with reporting structures. Such coordination mechanisms should be further be embedded in operational and monitoring frameworks and guidelines, identifying clear progress indicators to track the effectiveness of multisectoral collaboration (e.g., the number of joint AMR action plans developed across ministries; frequency and attendance rates of intersectoral meetings; and achievement of jointly defined outcome targets).

### Foster trust and shared accountability across sectors

An important element of the target-setting process must be engaging multisectoral stakeholders such as healthcare professionals, government officials, and community partners to foster trust, and facilitate shared ownership across relevant One health sectors and stakeholders (2). The joint responsibility and a strong sense of ownership which are engendered through this collaborative process can support a sense of shared accountability among actors, and in turn, help reduce siloed policy responses by establishing a shared vision of anticipated collaboration outcomes.

### Promote inclusivity, transparency, and policy coherence

Participation is another central dimension as inclusive target development practices promote institutionalization of regular multisectoral stakeholder dialogues during target setting and promote transparency and inclusivity in policy formulation through evidence-informed decision-making frameworks. Such meaningful participation during target setting will further strengthen buy-in for other initiatives to tackle AMR. Inclusive participation can also promote policy coherence, and in turn facilitate effective allocation of human and financial resources needed to implement AMR strategies. This process should also include coordinating AMR actions and targets with other major national and global policy efforts, such as achievement of sustainable development goals (SDGs) as mainstreaming of AMR, and integrating it into wider national development challenges, becomes more important.

### Set clear AMR-specific and AMR-sensitive targets

Target setting should include both AMR-specific as well as AMR-sensitive targets. AMR-specific targets focus on limiting the spread of AMR by targeting their immediate drivers. This may include targets such as reducing inappropriate antibiotic use in humans and animals, strengthening surveillance of resistant pathogens, and ensuring access to quality-assured antimicrobials. AMR-sensitive targets address the broader and underlying socio-structural drivers of AMR, such as infrastructure weaknesses and social system dysfunction which influence AMR. AMR-sensitive targets may include targets such as access to water, sanitation and hygiene, strengthening health systems, and enhancing IPC in healthcare facilities. This collaborative process should involve identifying an achievable number of high-impact national AMR targets tied to national priorities, including identifying target related to sustainable development (e.g., reducing inappropriate antibiotic use in primary care, increasing IPC compliance in hospitals, or phasing-out growth promotion use of antibiotics in agriculture).

### Invest in context-appropriate surveillance systems

To allow the monitoring of targets, countries should also increase investment in integrated surveillance systems, as target setting without monitoring capacity will undermine AMR policy effectiveness. In resource-constrained settings with limited surveillance capacity, a pragmatic and incremental approach to target setting might be most appropriate, with an initial focus on leveraging proxy indicators to measure progress, for example, antibiotic sales and dispensing data, volume of antimicrobials used in food-producing animals, or number of health facilities submitting data to national AMR platforms. Lastly, establishing clear, achievable targets that are aligned with international standards is essential, including that targets are specific, measurable, achievable, relevant, and time-bound (SMART) to ensure they are actionable. Leveraging international support and aligning national policies with global AMR strategies can enhance the effectiveness of national efforts and ensure that national AMR target-setting efforts learn from previous experiences with and build on the best practices in AMR target setting (2).

### Links to global accountability and monitoring frameworks

The development of national AMR targets must take into consideration the emergence of a global accountability framework established through the 2024 High-Level Political Declaration on AMR, which includes a global target of reducing total deaths associated with bacterial AMR by 10% by 2030. This same declaration also establishes a broader set of measurable global ambitions, including increasing the proportion of antibiotics in human health that are drawn from the WHO Access group, strengthening financing and implementation of national action plans, and reducing inappropriate antimicrobial use across sectors. The WHO Access, Watch and Reserve (AWaRe) classification provides countries with an established framework for translating global antimicrobial use commitments into measurable national targets and indicators. National target-setting processes should seek not only to address country-specific priorities and contexts, but also to contribute to internationally agreed-upon global goals. In addition, national AMR targets could be linked to existing WHO Monitoring and Evaluation framework indicators and country reporting mechanisms, such as the Tracking AMR Country Self-assessment Survey (TrACCS), thereby ensuring that national progress contributes directly to global monitoring efforts. Mapping context-specific targets into existing core indicators could also help reduce duplication of data collection, enhance comparability across countries, improve accountability, and help avoid the development of aspirational targets that cannot be routinely measured or monitored. Finally, the feasibility and sustainability of AMR targets are closely linked to the availability of dedicated financing, workforce capacity, and effective coordination mechanisms, highlighting the importance of aligning target-setting processes with realistic assessments of implementation and measurement capacity, as well as resource needs.

## Conclusion

The integration of measurable targets within NAPs on AMR has in global policy discussions been identified as a catalyst to improve AMU and related AMR outcomes. We argued in this perspective that establishing SMART targets is essential for effective AMR mitigation, improving resource allocation, facilitating monitoring and evaluation, and enhancing accountability. We further suggest that global-level targets announced in the High-Level Political Declaration on AMR must be matched by national and local policy commitments that are context-specific and realistic but ambitious. Going forward, AMR target setting and monitoring should also be aligned with other relevant national policy and development processes, such as national efforts surrounding the Sustainable Development Goals which would help ensure that national AMR strategies and target-setting efforts are integrated into broader national development agendas and promote long-term, cross-sectoral sustainability.

## Data Availability

The original contributions presented in the study are included in the article/supplementary material, further inquiries can be directed to the corresponding author.
